# Accessory nipple over the right scapula of a 14-year-old boy: An extremely rare and unreported location, case report

**DOI:** 10.1016/j.ijscr.2018.12.007

**Published:** 2019-01-19

**Authors:** Ayad Ahmad Mohammed

**Affiliations:** University of Duhok, College of Medicine, Department of Surgery, Azadi Teaching Hospital, 8 Nakhoshkhana Road, 1014 AM, Duhok City, Iraq

**Keywords:** Accessory nipple, Supernumerary nipple, Ectopic breast, Polythelia, Polymastia, Embryonic milk line

## Abstract

**Introduction:**

Accessory or supernumerary nipples can be applied when more than 2 breasts present in human beings. Usually they are seen in the embryonic milk line; however, their presence outside this line is extremely rare.

A 14-year-old boy presented to the surgical consultation room complaining from a round brown skin lesion over the right shoulder area, there were no symptoms associated with this lesion but the family was worried about it. During examination the lesion was diagnosed on the basis of clinical examination and appearance as a supernumerary nipple located over the right scapula. This is an extremely rare location, and no case has been reported before. The family was reassured about the diagnosis that this condition is congenital and the patient discharged with no specific treatment.

**Conclusion:**

Accessory nipple is not uncommon, and may present in some rare locations. The main work up should be reassurance of the patient about the diagnosis, and most patients need no intervention however it may be removed for cosmetic concerns, if enlarge in size, or associated with any symptom.

## Highlights

• The nipples or breasts can occur along the so called milk line, between the armpit and anterior thigh.
• Breast neoplasm may develop in ectopically located glandular tissue.
• Accessory breast cancer is usually diagnosed by clinical examination and ultrasonography.

## 1 Introduction

Many terms had been used to describe the presence of this condition, like Supernumerary breast tissue, Polythelia, polymastia, ectopic breast tissue, and accessory nipple [[1], [2], [3], [4]].

This term can be applied when more than 2 breasts present in human beings [5].

The frequency has been estimated to be around 1/500 and usually seen in the anterior aspect of the trunk, in the embryonic milk line. Most lesions are smaller than the normal nipple appearing as small spots of 2–3 mm in diameter [6,7]. Cases has been reported in rare locations like the face [1].

This the work has been reported in line with the SCARE criteria [8].

## 2 Patient information

A 14-year-old boy presented to the surgical consultation room because his mother was worried about a round brown skin lesion over the right scapula which enlarged in size and became deeper in color over the last year, there were no symptoms associated with this lesion but the family was worried about it. There were no relevant past medical, past surgical, or family histories for the chronic illnesses or skin diseases.

### 2.1 Clinical findings

**Fig. 1:**
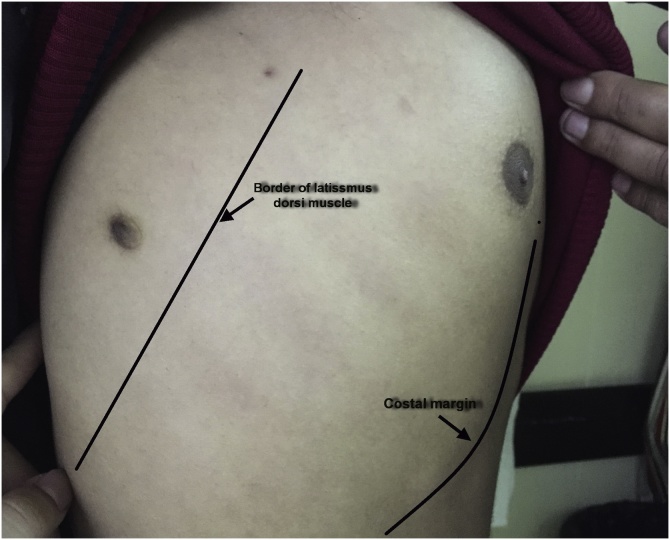
Showing the supernumerary nipple behind the anterior border of latissimus dorsi muscle, the costal margin is marked anteriorly with the normal right nipple shown.

During examination the lesion was a round, brown, and slightly elevated from the skin surface. The size was about 1 cm in diameter with central projection. The location of the mass was in the region of the right shoulder, Fig. 1.

### 2.2 Diagnostic assessment

On the basis of this clinical appearance the condition diagnosed as a supernumerary nipple located in this site.

This is an extremely rare location, and no case has been reported before.

### 2.3 Therapeutic intervention

No specific therapeutic work up done.

### 2.4 Follow-up and outcomes

The family was reassured about the diagnosis and the patient sent home to be followed if any symptom developed in the future.

## 3 Discussion

The supernumerary nipples are more common in males and most of them occur in the left side [9].

It may be familial and reported in many generations of some families [10,11].

If associated with excessive breast tissue it may become noticeable at puberty due to hormonal effect, or enlarge during pregnancy and lactation [4].

The diagnosis needs high index of suspicion, and sometimes in unclear situations may need histological confirmation which shows all the histological structures seen in the normal nipple like mammary glands and smooth muscles [2,6].

It may present as cosmetic concern and usually they are not associated with other congenital anomalies [2,12].

Accessory breasts may have the potential to develop the same benign and malignant disorder as normal breast tissue [5,13].

In most cases it may not need any intervention, however it may be removed surgically if there is cosmetic concern, uncertainty of the diagnosis, or the development of any kind of symptoms [4].

## Conflicts of interest

The author has no conflicts of interest to declare.

## Sources of funding

None.

## Ethical approval

This case reports not needed ethical approval.

## Consent

The father of the patient has provided me with a written informed consent to publish this finding of his son, and the identity of the patient has been protected.

## Author’s contribution

The concept of reporting the case, drafting, and revision of the case done by Dr Ayad Ahmad Mohammed.

## Registration of research studies

This work is case report and there is no need of registration.

## Guarantor

Dr Ayad Ahmad Mohammed is guarantor for the work.

## Provenance and peer review

Not commissioned, externally peer-reviewed.
